# Eye tracking technology in medical practice: a perspective on its diverse applications

**DOI:** 10.3389/fmedt.2023.1253001

**Published:** 2023-11-17

**Authors:** Mohammed Tahri Sqalli, Begali Aslonov, Mukhammadjon Gafurov, Nurmukhammad Mukhammadiev, Yahya Sqalli Houssaini

**Affiliations:** ^1^ Department of Economics, School of Foreign Services, Georgetown University in Qatar, Doha, Qatar; ^2^ Department of Engineering, New York University, Abu Dhabi, United Arab Emirates; ^3^ Department of Control and Computer Engineering, Polytechnic University of Turin, Turin, Italy; ^4^ Department of Business Administration, Carnegie Mellon University in Qatar, Doha, Qatar; ^5^ Department of Medicine, Faculty of Medecine and Pharmacy, Mohammed V University, Rabat, Morocco

**Keywords:** eye tracking, medical practice, medical training, human-computer interaction, diagnostic accuracy

## Abstract

Eye tracking technology has emerged as a valuable tool in the field of medicine, offering a wide range of applications across various disciplines. This perspective article aims to provide a comprehensive overview of the diverse applications of eye tracking technology in medical practice. By summarizing the latest research findings, this article explores the potential of eye tracking technology in enhancing diagnostic accuracy, assessing and improving medical performance, as well as improving rehabilitation outcomes. Additionally, it highlights the role of eye tracking in neurology, cardiology, pathology, surgery, as well as rehabilitation, offering objective measures for various medical conditions. Furthermore, the article discusses the utility of eye tracking in autism spectrum disorders, attention-deficit/hyperactivity disorder (ADHD), and human-computer interaction in medical simulations and training. Ultimately, this perspective article underscores the transformative impact of eye tracking technology on medical practice and suggests future directions for its continued development and integration.

## 1. Introduction

Eye tracking has emerged as a valuable technological tool in the field of medicine, revolutionizing research and clinical practice through the provision of insights into human behavior, cognition, and visual perception. By precisely measuring and analyzing eye movements, eye tracking enables researchers and medical professionals to unravel intricate patterns of visual attention, gaze fixation, and cognitive processes (1). Eye tracking has now become an integral component of medical research, facilitating investigations into various aspects of human behavior and cognition. Researchers employ eye tracking to unravel the complex mechanisms underlying visual attention, decision-making, and cognitive load within the medical practice (2). By tracking eye movements, researchers gain insights into gaze patterns, fixations, and saccades, which contribute to understanding the dynamics of human perception and information processing (3). Studies related to the field of eye tracking have significantly advanced other neighboring interdisciplinary fields including computing, human-computer interaction, psychology, and neuroscience (2). Together, these contributions enrich medical practice and medical outcomes. In this perspective article, we explore how eye tracking has proven particularly valuable from two main perspectives (1) the medical research domain as well as (2) the medical practice domain.

The article builds on top of the work of Zammarchi and Conversano (4) who initiated a bibliometric analysis on the applications of eye tracking in medicine. It provides a viewpoint from the medical research domain by showcasing how eye tracking has proven particularly valuable through different research applications. These applications include investigating visual perception impairments, cognitive deficits, and decision-making processes in patients with various medical conditions. Beyond the realm of research, the article explores how eye tracking finds extensive use in medical practice, revolutionizing clinical assessments and patient care. It also uncovers how eye tracking serves as a diagnostic tool, enabling clinicians to evaluate visual function and detect abnormalities in patients with neurological disorders and cognitive impairments. Moreover, the article showcases how eye tracking serves as a support tool in medical practice, providing valuable information to assist healthcare professionals in making informed decisions. This information can enhance surgical techniques, optimize workflow, and improve patient safety. We finally delve into the clinical applications of eye tracking in the field of medicine by highlighting numerous studies from various medical specialties. This allows us to demonstrate how deviations in eye movement measurements can be showcased. We shed the light on how abnormalities in eye movement metrics, such as reduced saccadic amplitude, prolonged fixation durations, or gaze deviations, have been identified as potential bio-markers for early detection and monitoring of these conditions. The following section discusses the three facets of applying eye-tracking within the medical practice, mainly serving as an enhancement tool for (1) diagnostic accuracy (2) assessment and improvement of medical performance (3) as well as the improvement of rehabilitation outcomes. Through these facets, this article aims to explore the diverse applications of eye tracking in medicine, focusing on its role in medical research, clinical practice, and its utility as both a decision support tool and a diagnostic tool.

## 2. Application facets for eye tracking in medical practice and research

### 2.1. Enhancing diagnostic accuracy

Eye tracking technology has shown promise in enhancing diagnostic accuracy in various medical domains. This section describes the utilization of eye tracking in specific medical conditions to improve diagnostic accuracy. We select examples in key medical fields that highlight this aspect. Among the selected fields we showcase the applications of eye tracking in ophthalmology, as well as neurology, including attention deficit hyperactivity disorder (ADHD) and autism spectrum disorder (ASD). As discussed by Clark et al. (5), there exists immense potential and value when eye tracking is used in the ophthalmology clinic. Eye tracking is employed in ophthalmology to aid in the diagnosis and monitoring of conditions such as glaucoma, macular degeneration, and diabetic retinopathy (5). For example, glaucoma patients display extended saccade response times which can be identified with the help of eye tracking (6). Furthermore, eye tracking can be more time-efficient and effective for diagnosing patients with myasthenia gravis (7). By analyzing eye movements and fixation patterns during visual tasks, clinicians can identify subtle abnormalities in visual function. Objective measurements provided by eye tracking enable the early detection of disease progression, contributing to improved diagnostic accuracy. In the field of neurology, eye tracking serves as a diagnostic tool for conditions including Parkinson’s disease and Alzheimer’s disease (4). Abnormal eye movement patterns, such as reduced saccadic velocity or impaired smooth pursuit, have been identified as potential biomarkers for these disorders. Bek et al. (8) used eye tracking to measure emotion recognition by people with Parkinson’s disease. The study concluded that eye movements reveal subtle effects of motion on emotion processing in Parkinson’s. Thus, eye tracking technology facilitates the objective measurement of these emotional abnormalities within people with early Parkinson’s disease symptom, leading to more accurate diagnoses. Another application of eye tracking is in the diagnosis of ADHD and ASD. As explained by Lev et al. (9), integrating eye tracking with continuous performance tests for ADHD is a feasible way of enhancing diagnostic precision. It also shows initial promise for clarifying the cognitive profile of ADHD patients. The results of the study by Lev et al. (9) show that individuals with ADHD often exhibit atypical patterns of visual attention, including increased distractibility or difficulties in maintaining focus. Through eye tracking, clinicians can objectively measure these attentional impairments, providing additional evidence for accurate diagnosis. A similar application of eye tracking in the identification of neurodevelopmental disorders is the diagnosis of ASD. According to a comprehensive literature review done by Papagiannopoulou et al. (10), eye tracking technology has proven valuable in diagnosing ASD especially among the childeren category. According to the review, individuals with ASD often exhibit differences in social attention, including reduced eye contact and atypical gaze patterns during social interactions. By objectively measuring these gaze behaviors, eye tracking aids clinicians in diagnosing ASD and distinguishing it from other developmental disorders.

### 2.2. Assessment and improvement of medical performance

The second key application facet for eye tracking in medical training and research is the improvement of medical performance. This can be either through a better diagnostic of medical images, or through improved medical procedures and operations. In this section, we discuss applications ranging from electrocardiogram (ECG) interpretation to surgical skill assessment. Eye tracking offers valuable insights into gaze behavior, visual attention, and decision-making processes. By leveraging these insights, clinicians and researchers can develop targeted interventions, optimize training programs, and enhance overall performance in rehabilitation and medical practice. The first example is the application of this technology on surgery. Eye-tracking systems significantly contribute to increased surgical performance and create new opportunities for enhancements in surgery. Gaze-based navigation and control enable interaction with computer interfaces or surgical robots by guiding surgeons’ gaze, creating a facilitation in hands-free control (11). As a consequence, there is a simplification of complex procedures and enhanced precision during surgeries to an order of magnitude. Moreover the authors (11) discuss examples where surgical instrument tracking and automation are made possible via the analysis of a surgeon’s gaze. Based on the surgeon’s gaze patterns, eye-tracking systems can predict which target or instrument the surgeon is intending to interact with, thereby automating certain actions, such as retraction or suction, based on the surgeon’s visual focus (12). Such automation feature of eye-tracking systems helps significantly reduce the amount of mental effort on surgeons. Eye-tracking technology is also used for cognitive workload assessment (12). It thus supports surgeons in decision-making procedures. Analyzing surgeons’ gaze patterns enables researchers to evaluate how much cognitive effort was required for certain surgical tasks, measuring the duration and intensity of mental workload, thus helping hospitals develop solutions to improve operational workflows.

Moreover, eye tracking technology emerges as a valuable tool in the assessment and improvement of medical performance in the fields where medical images interpretation is required. Among theses fields are pathology, and cardiology, where reading histopathology slides and ECGs respectively is a part of the medical diagnosis. Hence, eye tracking offers insights into the visual behavior and cognitive processes of pathologists and cardiologists (13). By capturing and analyzing eye movements during diagnostic tasks, eye tracking can be used to increase diagnosis accuracy, boost training techniques, and reveal specialists’ level of expertise. Pathologists and cardologists rely on their visual expertise to analyze histopathological slides and ECGs respectively to reach accurate diagnoses. With the help of eye tracking technology, medical practitioners’ gaze patterns, fixations, and saccades can be examined, providing valuable insights on their attentional focus and decision-making process. The studies by Sqalli et al. (13, 14) demonstrated that analyzing eye movement patterns while reading an ECG can serve as an indicator of practitioners’ attention while viewing an ECG. The study was able to comprehend individual variations in navigational strategies and analyze the connection between visual attention and diagnostic performance using this methodology. Both studies reveal how medical students (14) who learn how to read an ECG, as well as experience cardiologists (13) aquire their visual expertise. This process was documented by a change in their visual attention and gaze trends. This change was significant. By integrating eye tracking into cardiology practices, potential diagnostic inaccuracies or biases can be identified, which will lead to improvements in diagnostic accuracy. Such insights may be used to design training programs and decision-making systems that will reduce diagnostic errors and improve precision.

Eye tracking can also be used to study and quantify the visual expertise of cardiologists. The previously discussed cardiology studies (13, 14) also investigated the relationship between eye movements and visual expertise in cardiologists. According to the research, expert cardiologists had unique eye movement patterns that included shorter fixation times, more focused scan routes, and more frequent saccades. These patterns indicated more efficient information processing and higher visual expertise (13). Understanding and being able to objectively measure the visual strategies of experts can improve training programs, allowing for the development of more effective instructional methods and fostering the growth of expertise in cardiology and other medical fields. Eye tracking insights can thus guide the design and adaptation of medical training programs and facilitate the acquisition of the visual skills (13). These skills would be necessary for accurate and efficient diagnoses. By incorporating eye tracking feedback into training programs, novice practitioners can quickly advance to expert-level competence through utilizing efficient visual learning methods. Novices can learn efficient visual exploration strategies, that will lead to improved diagnostic accuracy by analyzing the gaze patterns and fixations of experts (14). Additionally, by incorporating eye tracking into virtual training settings, students may practice diagnosing digital ECGs and and other medical images using experienced eye movement patterns. Learning through the analysis of eye tracking patterns within medical student cohorts also contributes to an increased accuracy of ECG interpretation among them by identifying common diagnostic pitfalls, reducing errors, and increasing consistency in diagnoses (14).

### 2.3. Improving rehabilitation outcomes

The third and final section discusses how eye tracking technology offers valuable applications in improving rehabilitation outcomes across various medical domains. This includes cognitive rehabilitation and visual rehabilitation. By providing objective measurements of gaze behavior, eye tracking helps identify deficits, guide interventions, and optimize rehabilitation strategies. An example of the application of eye tracking in cognitive rehabilitation is the case described by Trojano et al. (15), whereby a young patient with closed head injury, who had dense amnesia as well as disabling neurological defects, used eye tracking supported technology to develop communication skills. After an extended period of inability to communicate, the patient went through an eye tracking based cognitive training with the aim of using a computerized eye-tracker system for voiceless communication. By analyzing the patient’s gaze behavior during cognitive tasks, clinicians gained insights into the patient’s attentional processes, cognitive load, and information processing. With the use of manchine learning pre-trained models, technologies can anticipate what the patient desires to communicate. The study (15) explains how eye tracking can provide objective measurements of gaze patterns, fixation duration, or visual attentional biases during specific cognitive exercises. It also showcases how improved cognitive function can contribute to better overall rehabilitation outcomes. Another aspect of rehabilitation that eye tracking can enable is visual rehabilitation for individuals with visual impairments. The study conducted by Saunier et al. (16) proposed a hybrid Virtual Reality (VR) interface using eye-tracking and Brain-Computer Interface with a gamified application for the rehabilitation of dyslexia. The study proves that by tracking eye movements, clinicians can assess visual scanning patterns, fixations, and saccades. This information guides the development of interventions aimed at improving visual exploration, enhancing visual attention, and promoting efficient use of residual vision in order to rehabilitate patient with dyslexia. Hence, the study serves as a successful example for how eye tracking-based feedback can be provided to individuals to improve their ability to locate and attend to visual stimuli.

## 3. Discussion

The facets elaborated in the previous section unveil how eye tracking technology is a versatile tool with diverse applications in the field of medicine. The application facets discuss the wide-ranging potential of eye tracking in improving diagnostic accuracy, contributing to the assessment and improvement of medical performance, and enhancing rehabilitation outcomes. The applications can be extended towards coaching students and trainees, medical research or medical practice. However, in this section, we acknowledge the limitations and ethical considerations associated with the technology to ensure a balanced understanding of its implications.

While eye tracking holds significant potential, it is essential to consider its limitations. The accuracy and reliability of eye tracking measurements can be influenced by factors such as calibration errors, head movements, and participant cooperation. Most of the studies showcased above, as well as the studies that we conducted relating to ECG interpretation mention one or more limitation that challenged the accuracy of eye tracking data collection and analysis (4, 13, 14). Moreover, the use of eye tracking technology in the medical setting may be limited by the availability of suitable hardware and software. The interpretation and analysis of eye tracking data requires expertise and standardized protocols to ensure meaningful results. Applying eye tracking to the medical setting poses its own unique risks. This is due to the potential influence of context and task-specific factors on gaze behavior within the medical environment. Eye tracking measurements may vary depending on the specific task, environment, and stimuli used. The level of engagement, familiarity with the technology, and individual differences can also influence gaze patterns. This is especially with patients whose disorder prevent them from concentration like the case of ASD (9) as well as ADHD (10). Hence, reducing the quality of the collected eye tracking data. Therefore, generalizing findings from one specific task or context to other scenarios should be done with caution.

Ethical considerations also come forward when using eye tracking technology in the medical domain. One important aspect is ensuring the participants’ privacy while collecting the data through several safety measures like obtaining informed consent. Eye tracking involves capturing sensitive information about individuals’ gaze behavior and visual attention. It is crucial to inform participants about the purpose, potential risks, and benefits of the study and obtain their voluntary consent, as it was noted by the majority of the studies discussed. Safeguards should be in place to protect the confidentiality of the data and ensure compliance with relevant privacy regulations. Additionally, issues related to data ownership and storage should be addressed. Eye gaze data can be considered personally identifiable information (17), and proper procedures should be implemented for data management, storage, and potential sharing. Data anonymization techniques can be employed to protect individual identities and maintain confidentiality. Moreover, the potential for bias in the interpretation and application of eye tracking data should be considered. Researchers and clinicians should be cautious about making assumptions or drawing conclusions solely based on eye tracking measurements. This is especially in a domain as critical as the medical domain. This was clearly noted in the systems designed for identifying patients with ASD (9) as well as ADHD (10). Therefore, medical interpretations should be made in conjunction with other clinical and diagnostic information. This is to avoid over-reliance on eye tracking data alone. Lastly, the accessibility and affordability of eye tracking technology can pose barriers to its widespread use in medical settings. The cost of eye tracking devices, software, and technical expertise required for implementation may limit its availability in certain healthcare contexts. Ensuring equitable access to eye tracking technology is essential to avoid exacerbating healthcare disparities. It is important to note that recently a handful of initiatives are set to provide freely available and open-source APIs for research and development. Among these are the SmartEye API for Quantitative Assessment of Oculomotor Abnormalities by Kumar et al. (18). Also, the SacLab toolbox for saccade analysis by Cercenelli et al. (19) and finally the generic PyGaze library, which is an open-source cross-platform toolbox for minimal-effort programming to design eye tracking experiments by Dalmaijer et al. (20).

## 4. Conclusion

To conclude, this perspective article shed the light on how eye tracking technology emerges as a powerful tool in medicine, facilitating research, clinical practice, and patient care. Through precise measurements and analysis of eye movements, eye tracking provides invaluable insights into human behavior, cognition, and visual perception. In medical research, it enables investigations into various aspects of human cognition and decision-making processes. In clinical practice, it serves as both a decision support tool and a diagnostic tool, assisting healthcare professionals in making informed decisions and aiding in the accurate diagnosis and monitoring of medical conditions. In this article, we highlighted the use of the technology in various medical fields including neurology, cardiology, pathology, surgery as well as rehabilitation. The integration of eye tracking technology into medical practice holds significant potential to enhance patient care, optimize treatment outcomes, and advance our understanding of human visual and cognitive processes. We also highlighted the limitations and ethical considerations of this technology. While eye tracking technology holds a great potential in the medical domain, it is important to consider its limiting ethical aspects. Addressing measurement errors, understanding the influence of context, and ensuring participant privacy are crucial for the responsible use of eye tracking technology. Additionally, considerations of data ownership, potential biases, and accessibility should be taken into account to maximize the benefits and minimize the potential risks associated with this technology. By navigating these limitations and ethical considerations, eye tracking can continue to contribute to advancements in medical research and practice.

## Data Availability

The original contributions presented in the study are included in the article/Supplementary Material, further inquiries can be directed to the corresponding author.
